# The Impact of Cardiovascular Risk Factors on the Course of COVID-19

**DOI:** 10.3390/jcm11082250

**Published:** 2022-04-18

**Authors:** Katarzyna Wilk-Sledziewska, Piotr Jan Sielatycki, Natalia Uscinska, Elżbieta Bujno, Mariusz Rosolowski, Katarzyna Kakareko, Rafal Sledziewski, Alicja Rydzewska-Rosolowska, Tomasz Hryszko, Edyta Zbroch

**Affiliations:** 1 Department of Internal Medicine and Hypertension, Medical University of Bialystok, 15-540 Bialystok, Poland; kat_wilk@wp.pl (K.W.-S.); piotr.sielatycki@gmail.com (P.J.S.); n.kukawka@wp.pl (N.U.); mlodawska.ela@gmail.com (E.B.); mariusz.rosolowski@umb.edu.pl (M.R.); 2 2nd Department of Nephrology and Hypertension with Dialysis Unit, Medical University of Bialystok, 15-276 Bialystok, Poland; katarzyna.kakareko@umb.edu.pl (K.K.); alicja.rosolowska@umb.edu.pl (A.R.-R.); tomasz.hryszko@umb.edu.pl (T.H.); 3 Department of Radiology, Medical University of Bialystok, 15-276 Bialystok, Poland; rafal.sledziewski@umb.edu.pl

**Keywords:** COVID-19, cardiovascular, obesity, smoking, hypertension, lipid profile, diabetes, risk factors, cardiology

## Abstract

Aim of the study: The aim of our review is to indicate and discuss the impact of cardiovascular risk factors, such as obesity, diabetes, lipid profile, hypertension and smoking on the course and mortality of COVID-19 infection. Background: The coronavirus disease 2019 (COVID-19) pandemic is spreading around the world and becoming a major public health crisis. All coronaviruses are known to affect the cardiovascular system. There is a strong correlation between cardiovascular risk factors and severe clinical complications, including death in COVID-19 patients. All the above-mentioned risk factors are widespread and constitute a significant worldwide health problem. Some of them are modifiable and the awareness of their connection with the COVID-19 progress may have a crucial impact on the current and possible upcoming infection. Data collection: We searched for research papers describing the impact of selected cardiovascular risk factors on the course, severity, complications and mortality of COVID-19 infection form PubMed and Google Scholar databases. Using terms, for example: “COVID-19 cardiovascular disease mortality”, “COVID-19 hypertension/diabetes mellitus/obesity/dyslipidemia”, “cardiovascular risk factors COVID-19 mortality” and other related terms listed in each subtitle. The publications were selected according to the time of their publications between January 2020 and December 2021. From the PubMed database we obtain 1552 results. Further studies were sought by manually searching reference lists of the relevant articles. Relevant articles were selected based on their title, abstract or full text. Articles were excluded if they were clearly related to another subject matter or were not published in English. The types of articles are mainly randomized controlled trial and systematic review. An additional criterion used by researchers was co-morbidities and age of patients in study groups. From a review of the publications, 105 of them were selected for this work with all subheadings included. Findings and Results: The intention of this review was to summarize current knowledge about comorbidities and development of COVID-19 infection. We tried to focus on the course and mortality of the abovementioned virus disease in patients with concomitant CV risk factors. Unfortunately, we were unable to assess the quality of data in screened papers and studies we choose because of the heterogenicity of the groups. The conducted studies had different endpoints and included different groups of patients in terms of nationality, age, race and clinical status. We decide to divide the main subjects of the research into separately described subtitles such as obesity, lipid profile, hypertension, diabetes, smoking. We believe that the studies we included and gathered are very interesting and show modern and present-day clinical data and approaches to COVID-19 infection in specific divisions of patients.

## 1. Introduction

**Figure 1:**
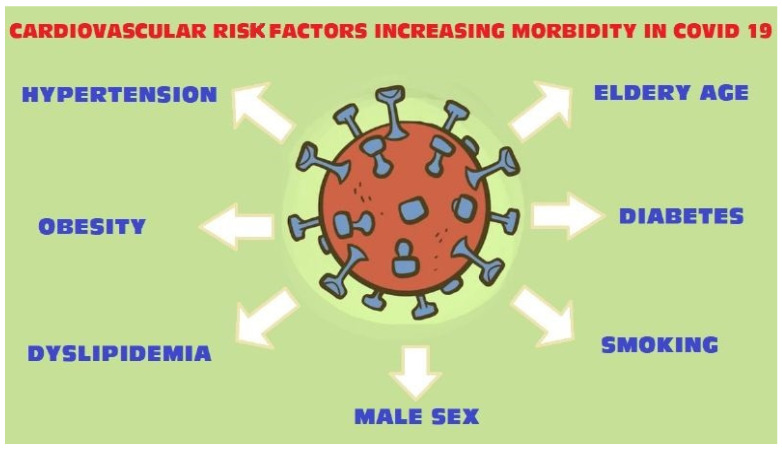
COVID-19 cardiovascular risk factors.

The coronavirus disease 2019 pandemic is spreading around the world and becoming a major public health crisis. All coronaviruses are known to affect the cardiovascular system. There is a strong correlation between cardiovascular risk factors and severe clinical complications including death in COVID-19 patients (Figure 1).

Diversified lifestyle, access to health care and prophylaxis, and an aging society contribute to the increasing number of patients suffering from civilization diseases such as obesity, hypertension, hyperlipidemia and diabetes. The presence of those comorbidities may deteriorate the course of COVID-19 infection.

Obesity was significantly associated with a higher in-hospital mortality as well as older age and male sex. Obese patients, <60 years old with Body Mass Index (BMI) ≥ 35 are 3.6 times more likely to be admitted to acute and critical care units, compared to patients with normal BMI in the same age [1].

Hyperlipidemia itself, as well as its treatment, contributes to the course of COVID-19 infection in a different way [2].

Hypertension is one of the most common and major cardiovascular risk factors. Worldwide, 1.33 billion people have elevated blood pressure. Chinese scientists who were the pioneers facing the COVID-19 infection indicated the connection between hypertension and higher risk of a severe course of COVID-19, including death [3].

Type 2 diabetes, especially with poorly controlled blood glucose, was found to be related to greater risk of acute respiratory distress syndrome (ARDS) development [4].

As coronavirus mostly affects the lungs, it is understandable that smoking, current and past, may be involved in the progress of COVID-19 [5].

The review aims to discuss the impact of mentioned cardiovascular risk factors on the course of coronavirus 2019 infection. They are widespread and constitute a significant worldwide health problem. Some of them are modifiable and the awareness of their connection with COVID-19 progress may have a crucial impact on the current and possible upcoming infection.

## 2. Obesity

### 2.1. Methods

We searched PubMed and Google Scholar databases, using the terms, “COVID-19 obesity”, “COVID-19 mortality obesity patients” as well as keywords such as “cardiovascular risk factors”, “obesity risk infections”. Further studies were sought by manually searching reference lists of the relevant articles. Relevant articles were selected based on their title, abstract or full text. Articles were excluded if they were clearly related to another subject matter or were not published in English. Out of 163 publications, we selected 20 for this subtitle.

### 2.2. Findings

Obesity, defined as BMI over 30 kg/m^2^ is associated with various disorders such as cardiovascular diseases, insulin resistance, type 2 diabetes mellitus, obstructive sleep apnea and some cancers [6,7]. It affects the immune response [8], endothelium imbalance [9], release of cytokines [10] and promotes chronic systemic inflammation [11]. All these features contribute to a worse course of infectious disease, prolonged hospitalization and worse outcomes in obese patients [12,13,14]. Therefore COVID-19 patients with obesity require particular attention.

In a study of 10,544 COVID-19 population, patients with a BMI of 30–40 kg/m^2^ had an increased risk for hospitalization and clinical deterioration compared to patients with a BMI below 30 kg/m^2^ [15]. In another study considering the COVID-19 infection, obesity together with age ≥ 52 years was strongly associated with illness severity [16]. Obesity was also shown as a high-risk factor for middle aged adult in a 3615 patients study. The authors suggested that obese patients aged between 52 and 60 years were more exposed to increased morbidity rates compared to patients > 60 years old [1]. What is more interesting, is there are studies that have reported the relation between poor prognosis of obese COVID-19 patients and gender. In a study by Cai Q et al. [17], the increased disease severity was correlated with the male sex. Similarly, Chiumello D. [18] found strong association between acute respiratory distress and male sex in overweight/obese patients. In contrast, a study of 32,583 patients indicated higher odds ratios in females than males. The authors suggested that females with obesity, diabetes and hypertension are more susceptible to COVID-19 and have a higher odds ratio for a severe COVID-19 course [19].

Obese patients more often demonstrated a cough and fever as initial symptoms, compared to normal weight patients [17]. Interestingly, in a small clinical study it was found that the increased area of visceral adipose tissue (measured at the level of the first lumbar vertebra on chest computed tomography) and upper abdominal circumference were associated with a higher probability of intensive care unit treatment or mechanical ventilation (adjusted for age and sex) [20]. There is also a strong association between a high BMI and mortality among the COVID-19 population. In a cohort of 20,133 cases, Docherty et al. [21] proved that obesity was significantly associated with a higher in-hospital mortality as well as older age, male sex and comorbidities such as chronic cardiac disease, chronic pulmonary disease, chronic kidney disease and liver disease. Further data from a Mexican study with 4103 COVID-19 cases showed a significant increase in hospitalization and mortality rate in patients with obesity [22]. Likewise, numerous clinical studies confirm the influence of obesity on outcomes of SARS-CoV-2 infection [23,24].

## 3. Lipid Profile

### 3.1. Methods

We choose 19 from 59 publications, found in PubMed, using keywords: “lipid profile and COVID-19 infections”, “hyperlipidemia/dyslipidemia and COVID-19”, “statins COVID”. The works we used are mainly relevant reviews, original publications and the literature they contain. Non-English articles were excluded.

### 3.2. Findings

The lipid profile plays a key role in viral infection. The cholesterol membrane was found as an important component for pathogenic viruses entering host cells [25]. Hao Wang et al. [26] indicated that a high level of cholesterol in the cellular membranes of tissue enhanced the entry of the virus. The authors suggested that high cellular cholesterol indicates SARS-CoV-2 infectivity. The average cellular cholesterol level in the lung increases with age, thereby the number of viral entry points rises. When cholesterol is low, as in children, there are only a few entry points. In chronically ill patients, where the cellular cholesterol level is high (mostly due to age and chronic inflammation), all the angiotensin I converting enzyme 2 (ACE2) receptors are positioned for viral infectivity. However, blood sample analysis did not correlate with cholesterol levels in the tissue cell membranes. This is because the chronic inflammatory process prompts the inhibition of cholesterol efflux proteins in the peripheral tissue. In a study with infectious bronchitis coronavirus, it was demonstrated that reduction of cholesterol prevented the binding of coronavirus with the host cells [27]. In another study with porcine deltacoronavirus, the authors observed the pharmacological reduction of cellular or viral cholesterol might block virus attachment and internalization [28]. On the other hand, a clinical study in China showed lower serum lipid levels (total cholesterol, HDL-cholesterol and LDL-cholesterol) in patients with COVID-19 infection compared to healthy controls. It was noticed that cholesterol level continued to drop during the first few days of infection and then gradually rose. The authors suggested the lipid changes might be related to viral–host cell fusion and entry, and thus may indirectly indicate the effectiveness of the COVID-19 treatment regimens [29].

There is evidence suggesting statins influence the course of COVID-19 infection, which could be useful in treatment. Statins are cholesterol-lowering drugs that possess beneficial effects such as anti-thrombotic, immunomodulatory and anti-inflammatory functions [30]. As a result of controlling cytokine overexpression and modulating immune responses, statins may prevent ARDS and may reduce the incidence of cardiovascular complications in COVID-19 patients [31,32]. Statin treatment may block viral infectivity through inhibition of glycoprotein processing [33]. The SARS-CoV-2 main protease (Mpro), a key coronavirus enzyme, has been examined as a potential protein target to prevent infection expansion [34]. Željko Reiner et al. [2] indicated that statins could be SARS-CoV-2 Mpro inhibitors and may block entry of the virus into host cells.

There is also data indicating statins’ ability to upregulate ACE2 signaling pathways, which could mitigate the invasion of SARS-CoV-2 through the ACE2 receptor [35]. A high level of ACE2 in pulmonary endothelium was associated with reduced severity of ARDS [36]. Moreover, statins might counteract SARS-CoV-2-induced endothelitis in lungs by promoting endothelial repair and accelerate recovery from ARDS in COVID-19 patients [37,38]. Statins, through their anti-inflammatory effects, protect from the occurrence of plaque rupture and, therefore, reduce the risk of myocarditis and cardiac injury in COVID-19 patients [39,40].

Retrospective data showed a positive impact of statin use on mortality and in-hospital outcomes in the COVID-19 population. In a retrospective study of 2147 patients with COVID-19, the multivariate Cox model showed, after adjusting for age, gender, comorbidities, in-hospital medications and blood lipids, lower risk of mortality, acute respiratory distress syndrome or intensive care unit treatment in the statin group vs. the non-statin group [41]. Another study of 13,981 patients with COVID-19, among which 1219 received statins 28-day all-cause mortality was significantly lower than in the non-statin group [42]. On the other hand, in a meta-analysis of retrospective observational studies investigating the impact of previous statin use in COVID-19 patients, no significant reductions in either in-hospital mortality or COVID-19 severity were reported among statin users. However, such reductions were found after adjusting for confounding risk factors [43].

## 4. Hypertension

### 4.1. Methods

Twenty-six publications were used from 829 results found in the PubMed database, we used keywords such as: “risk factors COVID-19 mortality”, “hypertension COVID-19 mortality”, “renin-angiotensin system COVID-19 mortality”, “angiotensin-converting enzyme COVID-19”.

We searched for more articles by manually searching reference lists of relevant articles. Relevant articles were selected based on their title, abstract or full text. As in the previous sections, non-English works were rejected.

### 4.2. Findings

Hypertension is one of the most common and major cardiovascular risk factors. Worldwide, 1.33 billion people (upwards of 1 in 4 men and 1 in 5 women) have been diagnosed as hypertensive [44].

Hypertension was also found as the most common comorbidity in patients with COVID-19 [45,46]. According to a retrospective study consisting of 487 COVID-19 patients in the Zhejiang Province of China, the prevalence of hypertension was higher in the 49 severe cases than in the 438 mild cases (53.1% vs. 16.7%, *p* < 0.0001) [3].

Since the coronavirus pandemic outbreak, many theories have emerged linking hypertension therapy together with COVID-19 infection. After initially hypothesizing a positive relationship between a use of renin–angiotensin–aldosterone system (RAAS) inhibitors and risk of coronavirus disease 2019, more recent evidence suggests negative associations [47,48]. There is no clear evidence that RAAS antagonists lead to up-regulation of ACE2 in human tissues [49,50,51].

The BRACE CORONA trial [52] showed no significant difference in the mean number of days alive and out of the hospital for those assigned to discontinue vs. continue angiotensin-converting enzyme inhibitors (ACEI) or angiotensin II receptor blockers (ARB) during COVID-19 infection. Those findings do not support routinely discontinuing ACEI or ARB among patients hospitalized with mild to moderate COVID-19 if there is an indication for the treatment.

The HFSA (Heart Failure Society of America), ACC (American College of Cardiology) and AHA (American Heart Association) recommended the continuation of ACEI/ARB drugs for those patients who were currently treated with such agents [50]. This statement was supported by studies which indicated the beneficial influence of RAAS antagonists. In an observation of 3017 patients with COVID-19, 1584 (52.5%) suffered from hypertension [51]. The mortality rate was lower among patients who were treated with ACEI (27%) or ARB (23%) compared to other antihypertensive drugs (39%) [51]. Similar findings were indicated in a multicenter clinical study, where 511 patients with COVID-19 and hypertension were included [53]. The studied population was divided into six groups: ACEI, ARB, CCB (calcium channel blockers), BB (beta-blockers), thiazide or none, depending on the medications taken. The treatment with ARB before hospitalization was found to reduce the risk of a severe course of the COVID-19 disease [53].

Patients with COVID-19 were often accompanied by electrolyte disturbances, due to SARS-CoV-2 binding to angiotensin I converting enzyme 2 and causing prevalent hypokalemia [54]. Therefore, the effect of ACEI/ARB on the potassium concentration may have a beneficial effect in these patients. Further, a few data showed that treatment with calcium channel blockers, widely used in hypertension patients, has a good effect on the course of COVID-19 infection [55]. Authors reported CCB can inhibit the replication of SARS-CoV-2 in vitro. They observed that taking amlodipine significantly reduced the death rate among COVID-19 patients. The results of this study are very promising but require further careful research.

Apart from the effects of antihypertensive drugs, the influence of hypertension itself on the course of COVID-19 was repeatedly observed. Yingyu Chen et al. [56] carried out the meta-analyses showing that COVID-19 patients with hypertension had higher mortality risk (OR = 2.3–95% CI (1.76, 3.00), *p* < 0.01). Similarly, Bo Li et al. [57] indicated a high blood pressure as a significant risk factor of a severe course of COVID-19. The incidents of hypertension were two folds higher in severe cases/ICU (intensive care unit) than in non-severe cases/ICU. Analogously, a meta-analysis on 1527 COVID-19 patients reported higher incidences of cardiovascular disease (three-fold), hypertension (two-fold) and diabetes (two-fold) among those who required intensive care unit admission compared to non-ICU patients [57]. Also, the hypertensive patients were more likely to develop ARDS (25). The study of 476 COVID-19 patients from three Chinese hospitals accentuated that the incidence of cardiovascular comorbidities, including hypertension was higher in those with severe and critical manifestations compared to those with a moderate clinical symptom [58]. Another meta-analysis indicated high blood pressure as one of the major comorbidities, which increased the risk of death from COVID-19 [59]. Wang B et al. [60] confirmed the higher risk of disease exacerbation in patients with hypertension. Interestingly, a notably higher risk was observed in patients under the age of 65 with hypertension, diabetes, cardiovascular diseases and cancer. The authors did not show a negative effect of taking antihypertensive drugs on COVID-19 infections.

It was also indicated that the immune system did not function properly in high blood pressure and SARS-CoV2 infection [61,62]. Jong-Chan Youn et al. [63] demonstrated concomitant lymphocyte CD8+ dysfunction in patients with hypertension. Therefore, lymphocytes were unable to effectively fight viral infection. For this reason, blood pressure should be properly controlled using pharmacotherapy. A very interesting analysis was also conducted among 287 black patients [64]. Researchers examined the association between hypertension, obesity and diabetes, individually and clustered as metabolic syndrome and COVID-19 outcomes in patients hospitalized during the peak of the pandemic. Only metabolic syndrome was associated with mortality, but not hypertension itself.

On the other hand, another analysis [65] showed no clear strong evidence of hypertension as an independent risk factor of COVID-19 and the authors did not suggest a deleterious effect of ACEI/ARB in COVID-19 infection. Similarly, in a multicenter retrospective Italian CORIST Study (the COVID-19 RISK and Treatments Collaboration) [66] authors evaluated a cohort comprising 3894 patients with COVID-19 in 30 clinical centers. Half of them had hypertension. It turned out that hypertension was not associated with increased in-hospital mortality as opposed to history of or active cancer, chronic degenerative diseases, previous myocardial infarction and obesity. Furthermore, among women with hypertension and patients hospitalized during the second wave of the pandemic, the mortality was even lower.

Despite various conclusions concerning both the hypertension itself and drugs used in hypertension, this cardiovascular risk factor has a significant impact on COVID-19 course but further studies are needed.

In the COVID-19 era, the challenge is to achieve a target blood pressure control in a “New Normal” lifestyle, in which health care workers may have a reduced opportunity for in-person clinical examination of patients. The remote blood pressure monitoring system using telemedicine was introduced in achieving target levels.

We summarized several studies concerning hypotensive treatment in COVID-19 infection in Table 1.

## 5. Diabetes

### 5.1. Methods

Thirty publications from 484 findings in PubMed database publications were chosen. We used only two terms: “COVID-19 diabetes mellitus”, “COVID-19 diabetes treatment”. We mostly searched for more articles by manually searching reference lists of relevant articles. Relevant articles were selected based on their title, abstract, full text and connections to the main topic of this paper. As in the previous sections, non-English works were rejected.

### 5.2. Findings

Recent studies reported diabetes mellitus as a risk factor for increased COVID-19 disease severity and higher mortality. Potential mechanisms underlying worse outcomes in diabetic COVID-19 patients include chronic inflammation, impaired immune response, increased coagulation activity and potential direct pancreatic damage by SARS-CoV-2 [67]. The diabetic population is particularly more susceptible to bacterial, parasitic and viral infections [68]. SARS-CoV-2 infection leads to increased reactive oxygen species (ROS) production [69,70,71], activation of the renin–angiotensin–aldosterone system, which together may cause insulin resistance, hyperglycemia and vascular endothelial damage [72,73]. It was shown that hyperglycemia is an independent predictor of morbidity and mortality in patients with severe acute respiratory syndrome (SARS) [74]. Recent research also proved impaired glucose metabolism and hyperglycemia may increase SARS-CoV-2 replication [75], which leads to a severe course of infection, frequent hospitalizations and increased mortality [76,77,78]. In a multicenter retrospective study of COVID-19 patients who were not previously diagnosed with diabetes, it was shown that high fasting glucose level (≥7.0 mmol/L, 126 mg/dL) at admission was an independent predictor of increased 28-day mortality. Therefore, it is important to treat hyperglycemia in patients with severe states of COVID-19 [79].

Zhu et al. [4] in a study of 7300 individuals with COVID-19 revealed significantly higher mortality risk in patients with type 2 diabetes, especially those with poorly controlled blood glucose. Another study showed that diabetes was significantly associated with elevated risk of a severe course of the disease [80] and the development of ARDS [75,76,77,78]. Xu et al. [81] reported that comorbidity with diabetes was an important independent risk factor predicting acute kidney injury among COVID-19 patients. In Onder’s study [82], from 355 deceased patients, 126 patients had diabetes (35.5%). Glucose-lowering treatment was also shown to influence the prognosis of COVID-19.

Commonly used dipeptidyl peptidase 4 inhibitors (DPP4i) not only decrease blood level of glucose but also played a role in the immune system as a marker of activated T lymphocytes and a regulator of the chemokines expression [83,84], which may lead to increased infection risk. In a study of 305,415 diabetic patients, it was shown that those using DPP4i inhibitors were more susceptible for upper respiratory tract infections [85]. In a retrospective, observational cohort study of 717 patients, DPP4i treatment was associated with higher risk of ICU admission [86].

However, evidence from further clinical trials does not confirm the above theory [87].

Although DPP4 might bind to SARS-CoV-2 similarly to ACE2 [88], the role of DPP4 inhibitors in SARS-CoV2 infection is entirely unclear. In an in vitro study, treatment with DPP4 inhibitors such as sitagliptin, vildagliptin or saxagliptin did not block the entry of coronaviruses into cells [89]. In a retrospective case–control study, sitagliptin treatment was associated with reduced mortality, better clinical outcomes and a greater number of hospital discharges [90]. On the other hand, Fadini et al. [91] in a study of patients with diabetes mellitus hospitalized due to COVID-19 pneumonia and with pneumonia of other etiology showed that use of DPP4 inhibitors had no protective properties against the coronavirus. Therefore, further prospective randomized clinical trials are needed to investigate the role of DPP4 inhibitors in patients with diabetes mellitus and COVID-19.

Glucagon-like peptide 1 (GLP1) receptor stimulation influence immune function and inflammatory processes [92,93]. GLP1 analogues in patients with type 2 diabetes reduced the rate of major adverse cardiac events [94].

In a study of 2449 diabetic patients, metformin users were compared with non-users. In the results, metformin treatment was associated with a lower risk of death in patients hospitalized for COVID-19 [95].

Interestingly, Cariou B. et al. [96] noticed a correlation between routine statin treatment and increased mortality in diabetic patients hospitalized for COVID-19.

SGLT-2 inhibitors (Sodium-glucose Cotransporter-2 Inhibitors) might afford additional vital organ protection in the settings of COVID-19. High hopes were placed on SGLT-2 inhibitors. In DARE-19 (Dapagliflozin in Respiratory Failure in Patients with COVID-19), a phase-3 multi-national double-blind placebo-controlled randomized trial, 1250 patients (with or without diabetes) were enrolled. Those groups were randomized 1:1 to placebo or dapagliflozin group. The results indicated dapagliflozin did not significantly reduce organ dysfunction or death, or improve recovery compared with placebo among noncritically ill hospitalized patients with COVID-19 [97].

The COVID-19 pandemic is driving significant changes in the healthcare system and disrupting current best practices for diabetic limb preservation, leaving large numbers of patients without adequate care. Some authors support triage systems that help reduce hospital visits for non-fatal wounds, allocating patients with less severe problems to office visits or even telemedical care and remote monitoring. Calling on people to stay at home will most likely reduce the amount of physical exercise compared to usual daily routine. Patients with COVID-19 should furthermore be re-educated in recognize and handle diabetic ketoacidosis since infection is one of its most frequent triggers. Telemedicine and other innovative strategies could be a reasonable approach to mitigate the problem of uncontrolled diabetes at least partly.

## 6. Smoking

### 6.1. Methods

Six publications were used searching through various databases (as above PubMed, in which we found 17 results using keyword “COVID-19 tobacco smoking”) and using the Google search engine, we manually found the most interesting works based on the title, abstract, the entire text and content suitability of a study or paper, for this subtitle. As in the previous sections, non-English works were rejected.

### 6.2. Findings

Cigarette smoking is a well-known cardiovascular risk factor. Many analyses, systematic reviews and observational studies showed the relationship between smoking and the course of COVID-19. Firstly, tobacco smoking was shown to increase the risk of coronavirus infection itself, because it promoted coughing, sneezing and the virus-containing aerosol was transmitted to people and surfaces. Meta-analysis of previously published works indicated current and past smoking leads to a severe clinical form of COVID-19, more frequent intensive care unit admission and death [96].

In a study of 1099 Chinese COVID-19 patients, 16% of smokers required hospitalization in intensive care units or died, compared to 5% of patients who never smoked [97]. In another study, Liu et al. [98] reported smokers were more likely to have severe symptoms, worse course of the disease and the need for mechanical ventilation. Within two weeks of hospitalization, 27% of smokers worsened, compared to 3% of non-smokers. The mortality rate was also higher among cigarettes users. A very interesting hypothesis is that exposure to nicotine may increase the risk of neuroinfection in COVID-19 through the interaction with the receptor ACE2 [99].

Nevertheless, the low prevalence of smokers among COVID-19 hospitalized patients was partly because many smokers were misclassified as nonsmokers [100]. Some patients may still have the false impression that smoking is protective against COVID-19 [101].

## 7. Conclusions

COVID-19 clinical presentation is heterogeneous, ranging from asymptomatic to severe cases. It is associated with a severe disease course in about 23% of cases and mortality in about 6% of infected persons [102]. On the other hand, it leads to changes in the phenotype of cardiovascular patients, which makes new clinical challenges and possible therapeutic options [103].

In recent months, many studies have been published specifying the influence of various factors and comorbidities on the course of COVID-19. Some of them are even contradictory. Therefore, the specific factor that can lead to disease progression in COVID-19 patients still remains unknown, the specific correlation between comorbidity and patients with COVID-19 continues to be unclear and needs further research.

## Figures and Tables

**Table 1:** Studies on antihypertensive treatment in COVID-19.

Study	Compared Drugs/Study Drugs	Population	Results
Ip Andrew et al. medRxiv (2020) [51]	ACEI/ARBs	3017 patients with COVID-19, 1584 (52.5%) suffered from hypertension	Lower mortality in patients treated with ACEI (27%) or ARBs (23%) compared to other antihypertensive drugs (39%)
Liu Y., et al. medRxiv (2020) [53]	ACEI, ARB, CCB, BB, thiazide or none.	511 patients with COVID-19 and hypertension	Treatment with ARBs before hospitalization compared, reduced the risk of severe course of the disease COVID-19 (*p* = 0.025)
Zhang L., et al. medRxiv (2020) [55]	CCB	487 adult COVID-19 patients with hypertension, among these patients 44 received amlodipine	Inhibit the replication of SARS-CoV-2 in vitro. Amlodipine significantly reduced the death rate among COVID-19 patients. Case fatality rate decreased form 26,1% in non-amlodipine groups vs. 6,8% in amlodipine group
Xu J., et al. Frontiers of Medicine (2020) [47]	ACEI/ARBs	702 patients, 40 patients were receiving ACEI/ARB, 61 patients were taking medication other than ACEI/ARB	No statistically significant differences in in-hospital mortality (28% vs. 34%, *p* = 0.46), ICU admission (20% vs. 28%, *p* = 0.37) or invasive mechanical ventilation (18% vs. 26%, *p* = 0.31) between patients with or without ACEI/ARB. No association between chronic receipt of RAAS and severe outcomes of COVID-19.

Note: ACEI, angiotensin-converting enzyme inhibitors; ARBs, angiotensin II receptor blockers; CCB, calcium channel blockers; BB, beta-blockers.

## Data Availability

No new data were created or analyzed in this study. Data sharing is not applicable to this article.
